# Trends in Prevalence of Gout Among US Asian Adults, 2011-2018

**DOI:** 10.1001/jamanetworkopen.2023.9501

**Published:** 2023-04-21

**Authors:** Chio Yokose, Natalie McCormick, Na Lu, Sruthi Tanikella, Kehuan Lin, Amit D. Joshi, Laura M. Raffield, Erica Warner, Tony Merriman, John Hsu, Kenneth Saag, Yuqing Zhang, Hyon K. Choi

**Affiliations:** 1 Clinical Epidemiology Program, Division of Rheumatology, Allergy, and Immunology, Massachusetts General Hospital, Boston; 2 The Mongan Institute, Department of Medicine, Massachusetts General Hospital, Boston; 3 Arthritis Research Canada, Vancouver, British Columbia, Canada; 4 Channing Division of Network Medicine, Department of Medicine, Brigham and Women’s Hospital, Harvard Medical School, Boston, Massachusetts; 5 Regeneron Pharmaceuticals, Tarrytown, New York; 6 Department of Genetics, University of North Carolina, Chapel Hill; 7 Clinical and Translational Epidemiology Unit, Massachusetts General Hospital, Boston; 8 Harvard/MGH Center on Genomics, Vulnerable Populations, and Health Disparities, Boston, Massachusetts; 9 Division of Clinical Immunology and Rheumatology, the University of Alabama, Birmingham; 10 Department of Biochemistry, University of Otago, Dunedin, New Zealand; 11 Department of Health Care Policy, Harvard Medical School, Boston, Massachusetts

## Abstract

**Importance:**

Gout disparities among Black individuals in the US have recently been explained by socioclinical factors; however, no information is available among Asian individuals living in Western countries, despite their disproportionately worsening metabolic health.

**Objective:**

To determine the prevalence of gout and serum urate concentrations according to race and ethnicity and to explore the association of social determinants of health and clinical factors.

**Design, Setting, and Participants:**

This is a population-based, cross-sectional analysis. Data from a nationally representative sample of US adults were obtained from the National Health and Nutrition Examination Survey (NHANES) (2011-2018) in which Asian race data were collected (primary). Data from the UK Biobank (2006-2021) were used for replication of the Asian vs White differences. Data analysis was performed from December 2021 to September 2022.

**Main Outcomes and Measures:**

Race-specific gout prevalence and serum urate levels.

**Results:**

A total of 22 621 participants from NHANES (2011-2018) were included in the analysis (mean [SD] age, 49.8 [17.8] years; 10 948 male participants [48.4%]). In 2017 to 2018, gout affected 12.1 million US individuals, with its crude prevalence increasing from 3.6% (95% CI, 2.8%-4.5%) in 2011 to 2012 to 5.1% (95% CI, 4.2%-5.9%) in 2017 to 2018 (*P* for trend = .03); this trend was no longer significant after age adjustment (*P* for trend = .06) or excluding Asian individuals (*P* for trend = .11). During the same period, age- and sex-adjusted prevalence among Asian Americans doubled from 3.3% (95% CI, 2.1%-4.5%) to 6.6% (95% CI, 4.4%-8.8%) (*P* for trend = .007) to numerically exceed all other racial and ethnic groups in 2017 to 2018, with age- and sex-adjusted odds ratio (ORs) of 1.61 (95% CI, 1.03-2.51) and a socioclinical factor–adjusted multivariable OR of 2.62 (95% CI, 1.59-4.33) for Asian vs White individuals. The latest age- and sex-adjusted gout prevalence among US individuals aged 65 years and older was 10.0% among White individuals and 14.8% among Asian individuals (including 23.6% of Asian men). Serum urate concentrations also increased between 2011 and 2018 among US Asian individuals (*P* for trend = .009). The Asian vs White disparity was also present in the UK Biobank.

**Conclusions and Relevance:**

The findings of this study suggest that the prevalence of gout among Asian individuals numerically surpassed that for all other racial and ethnic groups in 2017 to 2018. This Asian vs White disparity did not appear to be associated with socioclinical factors.

## Key Points

**Question:**

Does the prevalence of gout differ between Asian and White adults in the US, and are social and clinical factors associated with the variation?

**Findings:**

In this cross-sectional study of 22 621 participants, gout was estimated to affect 12.1 million US adults in 2017 to 2018, with age- and sex-adjusted prevalence among Asian individuals doubling from 3.3% (in 2011-2012) to 6.6% (in 2017-2018) to numerically exceed other racial and ethnic groups. Excess gout prevalence existed among Asian vs White individuals and increased after adjustment for body mass index and socioclinical factors; similar disparities were replicated among Asian individuals in the UK.

**Meaning:**

These findings suggest that gout now affects more Asian individuals in the US than other racial or ethnic groups, and the disparity between Asian and White adults does not appear to be associated with socioclinical factors.

## Teaser

This cross-sectional study examines the prevalence of gout and serum urate concentrations according to race and ethnicity and explores the association of social determinants of health and clinical factors using data from the National Health and Nutrition Examination Survey, 2011 to 2018.

## Introduction

Gout leads to excruciatingly painful recurrent flares, with subsequently increased risk of myocardial infarction and stroke,^1^ and destructive arthritis if left untreated.^2^ It is the most common inflammatory arthritis, with a growing global disease burden^3,4^ and greater frequency in the US than in other Western countries.^5^ The overall prevalence of gout in the US had more than doubled between the 1960s, 1990s, and late 2008,^6^ and the latest prevalence was estimated to be 3.9% (9.2 million adults).^7^ Furthermore, there are racial and ecological disparities in gout disease burden.^7,8,9,10,11,12,13^ For example, a recent US nationwide study^13^ found that gout was more prevalent among Black individuals than White individuals, and this difference was entirely explained by social determinants of health and clinical factors.

However, no information is available among US Asian individuals, the fastest-growing racial and ethnic group in the US,^14^ but also one substantially underrepresented in US health research,^15^ or among their counterparts in other Western countries. The higher prevalence of *ABCG2*, *SLC2A9*, and other urate risk alleles^16^ and their disproportionately worsening trends and overall disparities in metabolic risk factors in Western countries such as the US^17,18,19^ and UK^20,21,22^ may play an interactive role. Yet, the available data indicate that gout prevalence in Asia has remained relatively low, particularly in countries from which most US Asian individuals originate (eg, 1.1% in China and Japan, 0.8% in South Korea, and 1.8% in Hong Kong).^5,23,24,25,26,27^ We aimed to determine the latest gout prevalence trends among Asian individuals in the US at the national level compared with other racial and ethnic populations. We then sought to replicate the Asian vs White differences using data from the UK Biobank (UKBB).

## Methods

### US National Health and Nutrition Examination Survey Study Population

The National Health and Nutrition Examination Survey (NHANES) assesses a representative sample of the noninstitutionalized US civilian population that is selected using a multistage, stratified sampling design. This analysis used data from the 4 latest cycles that collected responses on Asian race as a separate category^28^ and gout diagnoses: 2011 to 2012 (first with Asian-specific data), 2013 to 2014, 2015 to 2016, and 2017 to 2018. More information on the NHANES study population and procedures is provided in the eAppendix in Supplement 1. All procedures in each NHANES were approved by the National Center for Health Statistics ethics review board, and written informed consent was obtained at the time of enrollment. This study followed the Strengthening the Reporting of Observational Studies in Epidemiology (STROBE) reporting guideline for cross-sectional studies.

### Race and Ethnicity in NHANES

Race and ethnicity were based on self-report and were categorized as non-Hispanic White (hereafter referred to as White), non-Hispanic Black or African American (hereafter referred to as Black), Hispanic (Mexican and non-Mexican Hispanic), non-Hispanic Asian (persons having origins in any of the original peoples of East Asia, Southeast Asia, or Indian subcontinent, hereafter referred to as Asian), and other (eg, American Indian, Alaska Native, Native Hawaiian, Pacific Islander, >1 race, or any other race). More information on race and ethnicity data in the NHANES is provided in the eAppendix in Supplement 1.

### Gout and Hyperuricemia in NHANES

During the home interviews, all subjects were asked, “Has a doctor or other health professional ever told you that you had gout?” The measurement of serum urate in the NHANES 2011 to 2018 has been described elsewhere,^29,30^ as have details of quality control procedures.^31^ Our primary definition of hyperuricemia was a serum urate (ie, uric acid) concentration of greater than 7.0 mg/dL (to convert to millimoles per liter, multiply by 0.0595) among male patients and greater than 5.7 mg/dL among female patients, consistent with prior studies.^7,32^

### Covariates in NHANES

Covariates included sex, age, race, ethnicity, educational attainment, household size and income, health insurance, recent dietary intake, medical history, and use of prescription medications within the past 30 days (including diuretics), body mass index (BMI; calculated as weight in kilograms divided by height in meters squared), poverty status, alcohol consumption, Dietary Approaches to Stop Hypertension^33,34,35^ diet quality score, and chronic kidney disease status. More information on the collection and derivation of covariates is provided in the eAppendix in Supplement 1.

### UKBB Study Population

The UKBB resource is a prospective cohort of more than 500 000 residents of the UK aged 40 to 69 years at enrollment (2006-2010).^36^ Participants provided blood samples and information on sociodemographics, diet, medical conditions, medications, and anthropometrics. Detailed information on the study population and assessment of race, gout, hyperuricemia, and covariates is provided in the eAppendix in Supplement 1.

### Statistical Analysis

Data analysis was performed from December 2021 to September 2022. All statistical analyses of the NHANES data were performed using survey commands of SAS statistical software version 9.4 (SAS Institute), incorporating sample weights and accounting for the clusters and strata of its complex study design^37^ to represent the total US civilian, noninstitutionalized population. We calculated the prevalence (percentage) of gout and mean serum urate concentrations in the US adult population for each cycle from 2011 to 2012 to 2017 to 2018 according to sex and race and ethnicity. We also examined the prevalence among those aged 65 years and older, given that gout is primarily a disease of older individuals.^7^ We used US Census data from 2017 to 2018 to standardize the prevalence estimate of each NHANES cycle. To investigate linear trends in gout and hyperuricemia prevalence and mean serum urate over time, we performed multivariable linear and logistic regressions within each racial and ethnic group, with survey cycles as a continuous independent variable in the models, as done previously^18^ using the 2011 to 2012 cycle as the referent. We then evaluated race-by-time interactions using a logistic regression model including race and ethnicity, cycle, and race and ethnicity–by-cycle interaction term, with US Asian individuals as the reference group. We also conducted the trend analyses for each covariate using the 2011 to 2012 cycle as the referent. To report the latest population estimates for gout and hyperuricemia (in millions) for the most recent available cycle (2017-2018), we used totals from the American Community Survey or Current Population Survey, as per the NHANES analytic guidelines.^37^

We examined how the temporal trends in gout prevalence and serum urate concentrations within Asian individuals changed with the addition of each covariate (with a significant trend) in regression models. We then determined how the racial differences between Asian and White US individuals in gout prevalence and serum urate concentrations in the 2017 to 2018 cycle changed with the addition of prespecified covariates to the age- and sex-adjusted model, as recently done for disparities among Black US individuals.^13^ We started with social factors (low education [high school or less], poverty, and health insurance), then consecutively added alcohol, diet score, and BMI (3 lifestyle factors potentially associated with education and poverty), and finished with diuretics and chronic kidney disease status (clinical factors potentially associated with the other purported risk factors).

Similar analyses were conducted with the UKBB data, as detailed in the eAppendix in Supplement 1. All *P* values were 2-sided, and the significance level was set at *P* < .05.

## Results

### Trends in Gout Prevalence in the US Between 2011 and 2018

A total of 22 621 participants from NHANES 2011 to 2018 were included in the analysis (mean [SD] age, 49.8 [17.8] years; 10 948 male participants [48.4%]). Between 2011 and 2018, the overall crude prevalence of gout increased from 3.6% (95% CI, 2.8%-4.5%; 8.1 million individuals) in 2011 to 2012 to 5.1% (95% CI, 4.2%-5.9%; 12.1 million individuals) in 2017 to 2018 (*P* for trend = .03) (Table 1 and eTable 1 in Supplement 1). These prevalences were 3.5% and 4.8%, respectively, after age adjustment (*P* for trend = .06); additional adjustment for sex did not affect the result materially (*P* for trend = .06) (Table 1).

**Table 1.** Trends in Gout Prevalence, National Health and Nutrition Examination Survey, 2011-2018^a^

Variable	Prevalence, % (95% CI)				*P* value for trend
	2011-2012	2013-2014	2015-2016	2017-2018	
Overall	3.6 (2.8-4.5)	4.0 (3.2-4.9)	3.9 (3.2-4.5)	5.1 (4.2-5.9)	.03
Age adjusted	3.5 (2.7-4.3)	4.0 (3.1-5.0)	3.7 (3.1-4.23)	4.8 (3.9-5.7)	.06
Age and sex adjusted	3.5 (2.8-4.3)	4.1 (3.1-5.0)	3.8 (3.1-4.1)	4.8 (4.0-5.7)	.06
All participants^b^					
Asian^c^	3.3 (2.1-4.5)	2.8 (1.8-3.8)	4.1 (2.9-5.3)	6.6 (4.4-8.8)	.007
Hispanic	1.7 (1.0-2.5)	2.3 (1.6-3.0)	2.7 (1.8-3.6)	3.9 (2.2-5.5)	.03
Non-Hispanic Black	5.1 (4.0-6.2)	5.0 (3.5-6.5)	5.8 (4.7-6.9)	5.7 (4.4-7.1)	.50
Non-Hispanic White	3.7 (2.6-4.7)	4.3 (3.0-5.5)	3.6 (2.5-4.6)	4.9 (3.7-6.1)	.32
Other^d^	2.8 (0-6.12)^e^	5.2 (1.6-8.8)	6.3 (3.2-9.4)	3.5 (1.8-5.2)	.11
Male participants^f^					
Asian^c^	5.9 (3.9-7.9)	4.7 (2.8-6.7)	5.5 (3.1-7.9)	11.0 (6.7-15.2)	.05
Hispanic	2.5 (1.0-3.9)	4.0 (2.5-5.5)	3.4 (1.9-5.0)	5.4 (3.1-7.8)	.07
Non-Hispanic Black	5.9 (4.1-7.6)	6.2 (4.3-8.2)	8.6 (6.5-10.7)	8.3 (5.9-10.6)	.05
Non-Hispanic White	4.9 (3.7-6.1)	6.7 (4.9-8.4)	4.7 (3.3-6.1)	7.0 (4.9-9.0)	.38
Other^d^	5.3 (0-12.3)^e^	8.6 (2.1-15.0)	9.1 (4.2-14.1)	5.2 (2.1-8.3)	.24
Female participants^f^					
Asian^c^	0.9 (0.0-1.7)	0.9 (0.4-1.5)	2.8 (0.8-4.8)	2.4 (1.3-3.6)	.005
Hispanic	1.1 (0.3-1.9)	0.7 (0.1-1.2)	2.0 (1.2-2.9)	2.4 (0.9-3.9)	.08
Non-Hispanic Black	4.4 (2.6-6.2)	3.9 (1.7-6.1)	3.2 (2.2-4.2)	3.4 (2.1-4.7)	.17
Non-Hispanic White	2.5 (1.1-3.9)	2.0 (0.8-3.2)	2.5 (1.3-3.7)	3.0 (1.7-4.2)	.52
Other^d^	0.6 (0-1.7)^e^	2.1 (0-5.5)^e^	3.7 (1.5-56.0)	1.9 (0.2-3.6)	.41

^a^ Data are presented incorporating sample weights and adjusted for clusters and strata of the complex sample design of National Health and Nutrition Examination Survey 2011-2018.

^b^ Refers to age- and sex-standardized prevalence.

^c^ Asian race encompassed persons having origins in any of the original peoples of East Asia, Southeast Asia, or Indian subcontinent, including Cambodia, China, India, Japan, Korea, Malaysia, Pakistan, the Philippine Islands, Thailand, and Vietnam.

^d^ Other refers to American Indian, Alaska Native, Native Hawaiian, Pacific Islander, more than 1 race, or any other race.

^e^ Confidence intervals were derived according to binomial distribution for standardization; all negative values have been replaced with 0.

^f^ Refers to age-standardized prevalence.

During the same period, the age- and sex-adjusted prevalence among Asian adults doubled from 3.3% (95% CI, 2.1%-4.5%) to 6.6% (95% CI, 4.4%-8.8%) (*P* for trend = .007) to numerically surpass all other racial groups in 2017 to 2018 (Black, 5.7%; White, 4.9%; and Hispanic, 3.9%) (Table 1 and Figure). The rate of increase among Asian individuals was greater than that among White individuals (*P* for trend = .04) or Black individuals (*P* for trend = .047) (Figure). When Asian individuals were excluded from the overall sample, the temporal trend was no longer significant (*P* for trend = .11). Among Asian individuals, both women and men had an increase in age-adjusted gout prevalence (women: from 0.9% in 2011-2012 to 2.4% in 2017-2018; *P* for trend = .005; men: from 5.9% in 2011-2012 to 11.0% in 2017-2018; *P* for trend = .05), although it was not significant for men (Table 1). A similar trend was observed among Hispanic men and women and Black men, whereas the age-adjusted prevalence among White men and women remained stable (*P* for trend = .32) (Table 1).

**Figure.**
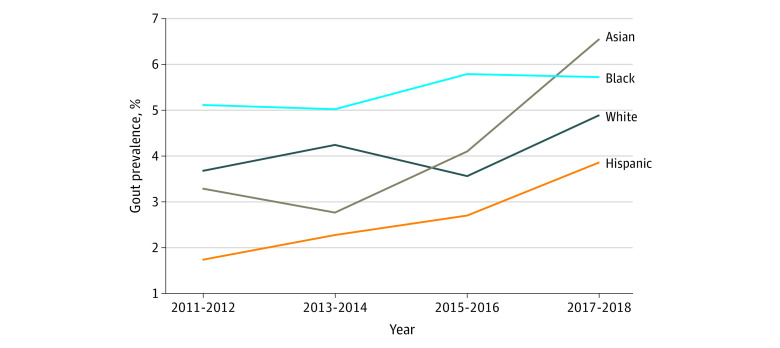
Trends in Gout Prevalence by Race and Ethnicity, 2011-2018 Graph shows age- and sex-standardized prevalence of gout among different US racial and ethnic groups. Data are presented incorporating sample weights and adjusted for clusters and strata of the complex sample design of National Health and Nutrition Examination Survey, 2011 to 2018.

The overall crude prevalence among older Americans (aged ≥65 years, Medicare-eligible age) increased numerically from 8.9% in 2011 and 10.5% in 2018, although the overall trend was not significant (*P* for trend = .29). However, the age- and sex-adjusted prevalence among older Asian individuals doubled from 7.7% to 14.8% (*P* for trend = .04) to numerically exceed all other racial groups in 2017 to 2018 (Black, 12.1%; Hispanic, 11.7%, and White, 10.0%) (Table 2 and eFigure in Supplement 1). The increase was also significant among older Hispanic adults (*P* for trend = .03). When stratified by sex, the age-adjusted prevalence among Asian men was 23.6% in 2017 to 2018, followed by Hispanic men (15.4%), Black men (14.3%), and White men (12.5%).

**Table 2.** Trends in Gout Prevalence by Race and Ethnicity Among Medicare Beneficiaries Aged 65 Years and Older National Health and Nutrition Examination Survey, 2011-2018^a^

Variable	Prevalence, % (95% CI)				*P* value for trend
	2011-2012	2013-2014	2015-2016	2017-2018	
Overall	8.9 (6.1-11.8)	7.9 (5.8-9.9)	8.5 (6.5-10.4)	10.5 (8.3-12.7)	.29
Age and sex adjusted	8.3 (5.6-11.0)	8.4 (5.5-11.2)	8.5 (6.1-10.8)	10.4 (7.9-13.0)	.26
All participants^b^					
Asian^c^	7.7 (2.3-13.2)	8.4 (2.1-14.7)	9.7 (3.4-15.9)	14.8 (8.3-21.4)	.04
Hispanic	3.1 (0.6-5.6)	5.4 (2.0-8.9)	7.3 (4.8-9.7)	11.7 (4.1-19.3)	.03
Non-Hispanic Black	14.6 (7.9-21.3)	15.5 (9.2-21.8)	16.5 (11.5-21.5)	12.1 (7.1-17.1)	.47
Non-Hispanic White	8.2 (4.9-11.4)	7.9 (4.9-10.8)	6.9 (4.1-9.6)	10.0 (7.1-13.0)	.56
Other^d^	6.5 (0-16.5)^e^	10.1 (0-22.9)^e^	24.6 (14.1-35.0)	8.1 (0-16.7)^e^	.21
Male participants^f^					
Asian^c^	11.9 (0.8-23.1)	13.7 (2.7-24.6)	9.2 (2.5-16.0)	23.6 (12.8-34.4)	.07
Hispanic	4.9 (0-10.0)^e^	8.8 (1.2-16.4)	8.6 (5.9-11.4)	15.4 (4.2-26.6)	.05
Non-Hispanic Black	14.9 (7.5-22.3)	20.2 (9.4-31.0)	25.0 (14.3-35.7)	14.3 (6.8-21.9)	.98
Non-Hispanic White	9.0 (4.1-13.8)	12.7 (5.5-19.9)	6.9 (4.3-9.8)	12.5 (7.4-17.7)	.68
Other^e^	14.3 (0-36.1)^e^	10.5 (0-27.0)^e^	44.9 (23.66-6.1)	15.2 (0-32.6)^e^	.24
Female participants^f^					
Asian^c^	4.0 (0-9.3)^e^	3.8 (0.1-7.5)	9.9 (0-20.5)^e^	7.3 (0.5-14.1)	.31
Hispanic	1.5 (0-4.2)^e^	2.4 (0-6.5)^e^	6.2 (2.9-9.5)	8.4 (1.7-15.2)	.08
Non-Hispanic Black	14.3 (5.1-23.6)	11.4 (2.8-20.0)	9.2 (6.5-11.8)	10.3 (3.8-16.7)	.34
Non-Hispanic White	7.5 (2.8-12.3)	3.62 (1.1-6.1)	6.94 (3.2-10.7)	7.89 (3.3-12.4)	.71
Other^d^	2.0 (0-13.7)^e^	10.0 (0-29.2)^e^	7.2 (1.9-12.5)	2.0 (0-6.5)^e^	.46

^a^ Data are presented incorporating sample weights and adjusted for clusters and strata of the complex sample design of National Health and Nutrition Examination Survey 2011-2018.

^b^ Refers to age- and sex-standardized prevalence.

^c^ Asian race encompassed persons having origins in any of the original peoples of East Asia, Southeast Asia, or Indian subcontinent, including Cambodia, China, India, Japan, Korea, Malaysia, Pakistan, the Philippine Islands, Thailand, and Vietnam.

^d^ Other refers to American Indian, Alaska Native, Native Hawaiian, Pacific Islander, more than 1 race, or any other race.

^e^ Confidence intervals were derived on the basis of binomial distribution for standardization; all negative values have been replaced with 0.

^f^ Refers to age-standardized prevalence.

### Trends in Mean Serum Urate in the US, 2011-2018

Among US adults in aggregate, the overall mean serum urate remained stable between 2011 and 2018 (eTables 1 and 2 in Supplement 1). However, after adjusting for age and sex, Asian individuals showed significantly increasing mean serum urate concentrations, from 5.4 mg/dL to 5.6 mg/dL (*P* for trend = .009), to become the numerically highest, together with Black individuals in 2017 to 2018 (Asian vs White, *P* for trend < .001; Black vs White, *P* for trend = .01).

### Covariate Adjustment and Trends Among Asian Individuals, 2011-2018

Among potential risk factors for the increasing trends for gout among Asian individuals, BMI and active insurance coverage increased significantly between 2011 and 2018, whereas other factors remained stable (eTable 3 in Supplement 1). When BMI and active insurance were both adjusted, the odds ratio (OR) per NHANES cycle attenuated from 1.39 (95% CI, 1.12-1.72) to 1.32 (95% CI, 1.07-1.63), and the OR comparing the latest NHANES cycle (2017-2018) to the earliest cycle (2011-2012) attenuated from 2.30 (95% CI, 1.32-4.01) to 2.09 (95% CI, 1.18-3.70) (Table 3). Adjusting for BMI nullified the serum urate trend (Table 3).

**Table 3.** Covariate Adjustment and Temporal Trends Among US Asian Individuals in Serum Urate Concentrations and Gout Prevalence, National Health and Nutrition Examination Survey, 2011-2018^a^

Biannual trends	Serum urate difference per biannual cycle, mean (95% CI), mg/dL	OR for gout per biannual cycle (95% CI)
Age and sex adjusted	0.07 (0.02 to 0.12)	1.39 (1.12 to 1.72)
Active health insurance	0.07 (0.02 to 0.12)	1.37 (1.11 to 1.69)
BMI^b^	0.03 (−0.02 to 0.07)	1.32 (1.07 to 1.63)
Dichotomous comparison: 2017-2018 vs 2011-2012 (Referent)		
Age and sex adjusted	0.24 (0.09 to 0.40)	2.30 (1.32 to 4.01)
Active health insurance	0.23 (0.08 to 0.39)	2.25 (1.30 to 3.90)
BMI^b^	0.11 (−0.04 to 0.26)	2.09 (1.18 to 3.70)

Abbreviations: BMI, body mass index; OR, odds ratio.

SI conversion factor: To convert urate (uric acid) to millimoles per liter, multiply by 0.0595.

^a^ Asian race encompassed persons having origins in any of the original peoples of East Asia, Southeast Asia, or Indian subcontinent, including Cambodia, China, India, Japan, Korea, Malaysia, Pakistan, the Philippine Islands, Thailand, and Vietnam.

^b^ BMI is calculated as weight in kilograms divided by height in meters squared.

### Disparity Between Asian and White Individuals in 2017 to 2018

In NHANES 2017 to 2018, the age- and sex-adjusted OR for gout among Asian individuals compared with White individuals was 1.61 (95% CI, 1.03-2.51), which increased after adjusting for social determinants of health, lifestyle, and clinical risk factors, particularly BMI, with a fully adjusted OR of 2.62 (95% CI, 1.59-4.33) (Table 4). Similarly, the age- and sex-adjusted excess in serum urate concentrations among Asian individuals compared with White individuals was 0.21 mg/dL (95% CI, 0.08-0.35 mg/dL), which increased with adjustment for socioclinical risk factors, particularly after BMI, with a fully adjusted difference of 0.50 mg/dL (95% CI, 0.37-0.62 mg/dL) (Table 4).

**Table 4.** Step-by-Step Regression for Potential Risk Factors for the Association Between Asian Race and Odds of Gout and Serum Urate Concentration Compared With White Individuals, NHANES 2017-2018 and UKBB^a^^,^^b^

Variable	NHANES 2017-2018 (US)		UKBB, 2006-2021	
	OR for gout (95% CI)	Serum urate concentration difference, mean (95% CI), mg/dL	OR for gout (95% CI)	Serum urate concentration difference, mean (95% CI), mg/dL
Age and sex adjusted	1.61 (1.03-2.51)	0.21 (0.08-0.35)	1.16 (1.05-1.29)	0.12 (0.10-0.15)
Education^c^	1.61 (1.03-2.52)	0.22 (0.08-0.36)	1.18 (1.07-1.32)	0.13 (0.10-0.15)
Poverty^d^	1.68 (1.07-2.63)	0.22 (0.08-0.36)	1.14 (1.02-1.26)	0.11 (0.09-0.13)
Active health insurance^e^	1.67 (1.07-2.63)	0.22 (0.08-0.36)	NA	NA
Alcohol consumption^f^	1.70 (1.08-2.68)	0.23 (0.09-0.37)	1.28 (1.14-1.42)	0.17 (0.15-0.19)
Dietary Approaches to Stop Hypertension diet score^g^	1.78 (1.11-2.83)	0.26 (0.13-0.39)	1.33 (1.19-1.48)	0.20 (0.18-0.23)
Body mass index^h^	2.37 (1.45-3.86)	0.46 (0.33-0.59)	1.64 (1.47-1.83)	0.31 (0.29-0.33)
Diuretic use^i^	2.37 (1.45-3.87)	0.47 (0.34-0.60)	1.64 (1.47-1.84)	0.30 (0.28-0.33)
Chronic kidney disease^j^	2.62 (1.59-4.33)	0.50 (0.37-0.62)	1.63 (1.46-1.82)	0.30 (0.28-0.33)

Abbreviations: NA, not applicable; NHANES, National Health and Nutrition Examination Survey; OR, odds ratio; UKBB, UK Biobank.

SI conversion factor: To convert urate (uric acid) to millimoles per liter, multiply by 0.0595.

^a^ Asian race in the NHANES encompassed persons having origins in any of the original peoples of the Far East, Southeast Asia, or Indian subcontinent, including Cambodia, China, India, Japan, Korea, Malaysia, Pakistan, the Philippine Islands, Thailand, and Vietnam. Asian ethnicity in the UKBB included Asian or Asian British, Chinese, Indian, Pakistani, Bangladeshi, or any other Asian background.

^b^ Estimates for each factor were generated from a sample with complete data on all variables.

^c^ Dichotomized as high school graduate or less vs some college or more.

^d^ Dichotomized as less than 1.3 (household income less than 130% of the federal poverty guideline) vs 1.3 or higher in (NHANES). In the UKBB, a continuous variable, the Townsend deprivation index, was used.

^e^ Active health insurance was not included in the UKBB models because of the universal publicly funded health care system in the UK.

^f^ In NHANES, alcohol consumption is a continuous variable (ie, number of drinks consumed per week). In the UKBB, it is a categorical variable (ie, frequency of alcohol consumption, with never as the reference).

^g^ In NHANES, this is a continuous variable (range, 9-45), with higher scores reflecting lower compliance with a Dietary Approaches to Stop Hypertension–style diet. In UKBB, it s a continuous variable (ie, mean servings per week of fish, poultry, and fish), and milk consumption is dichotomized as yes or no.

^h^ Body mass index is icalculated as weight in kilograms divided by height in meters squared. This is a continuous variable, and higher values are associated with greater adiposity.

^i^ Dichotomized as yes or no.

^j^ Dichotomized as estimated glomerular filtration rate less than 60 mL per minute per 1.73 m^2^ vs 60 mL per minute per 1.73 m^2 ^or higher.

### Disparity Between Asian and White Individuals in the UKBB

The overall prevalence of gout in the UKBB was 3.7%, and that of hyperuricemia was 15.7%. The age-standardized prevalence of gout was 3.3% among Asian individuals and 2.9% among White individuals, whereas the age-standardized prevalence of hyperuricemia was 16.0% among Asian individuals and 14.0% among White individuals (eTable 4 in Supplement 1). Among the 9826 Asian and 407 057 White participants included in the step-by-step regression analysis (eTable 5 in Supplement 1), the age- and sex-adjusted OR for gout comparing Asian with White individuals was 1.16 (95% CI, 1.05-1.29), which increased after adjustment for socioclinical factors, especially alcohol consumption and BMI, resulting in a fully adjusted OR of 1.63 (95% CI, 1.46-1.82) (Table 4). Similarly, the excess in serum urate concentrations among Asian participants compared with White participants increased from 0.12 mg/dL (95% CI, 0.10-0.15 mg/dL; age and sex adjusted) to 0.30 mg/dL (95% CI, 0.28-0.33 mg/dL; fully adjusted) (Table 4).

## Discussion

In this cross-sectional study of nationally representative US adults, we found the overall prevalence of gout increased between 2011 and 2018, and in 2017 to 2018 this painful condition affected 5.1% of adults (an estimated 12.1 million individuals). Adjusting for age attenuated the increasing trend, indicating that it was partially associated with the aging of the population. Among different racial and ethnic groups, the age- and sex-adjusted prevalence among Asian individuals doubled between 2011 and 2018, numerically surpassing all other racial groups to reach 6.6%. Correspondingly, mean serum urate concentrations also increased among Asian individuals during the same period, but this was nullified following adjustment for BMI. The Asian preponderance in 2017 to 2018 was especially large among Asian adults aged 65 years or older, with 14.8% affected, including 23.6% of older Asian men. The excess burden of gout among Asian adults was replicated in the UK, another prototypical Western country (although with modestly better metabolic health than the US^38^), and in both settings, these disparities strengthened after adjustment for BMI and socioclinical factors. This contrasts the Black vs White gout disparity in the US^13^ and likely reflects the combined effects of the higher prevalence of urate risk alleles and exposure to increasingly gout-prone environments among Asian individuals.

To our knowledge, this is the first study to examine national gout prevalence by race or ethnicity that specifically includes the prevalence among Asian individuals in Western countries. These findings are consistent with those from prior ecological data showing higher concentrations of serum urate and adiposity and poorer quality diet among Asian individuals living in the US than those living in East Asian countries.^39^ Recent reports indicate that the gout prevalence in countries from which most US Asian individuals originate remains low,^5,23,24,25,26,27^ although the frequency may be growing there as well.^23^ For example, the largest Asian origin group in the US is Chinese (24% of US Asian individuals),^40^ and gout prevalence in China is estimated to be 1.1%.^5^ Although potential genetic risk factors for gout have been identified in both Filipino^41^ and Vietnamese individuals,^42^ who comprise the third and fourth largest origin groups of US Asian individuals,^40^ gout prevalence in these countries still remains relatively low.^43,44^ Similarly, gout prevalences in Korea and Japan are 0.8%^26^ and 1.1%,^24^ with 7% and 9% of US Asian individuals originating from these respective countries.^40^ Although the prevalence in Taiwan has been estimated to be 6.2%, associated with indigenous population clusters genetically related to Polynesian and Oceania/Pacific Islander individuals (who have a known genetic predisposition to gout),^45^ less than 1% of the US Asian population is of Taiwanese origin.^40^ Among US populations, a prior study^46^ from Minnesota found a higher prevalence among Hmong than non-Hmong men (11.5% vs 4.1%), and a recent report^8^ of Medicare recipients in the Multiethnic Cohort (based in California and Hawaii) found a 14% higher risk of gout among older Japanese US participants, compared with older White participants, but did not study other Asian groups. Our national-level data show that Asian individuals now have the numerically highest prevalence of gout among US racial and ethnic groups, with a striking prevalence among older Asian men, among whom nearly 1 in 4 are affected by gout. These findings were also supported by elevated serum urate concentrations among US Asian individuals and replicated in the UKBB.

During the study period (2011-2018) in the US, Asian individuals have had disproportionate increases in BMI, waist circumference, and body fat percentage, compared with other US racial and ethnic groups.^18^ Among potential risk factors for the trends observed in our study, we confirmed a worsening BMI trend as well as increased insurance coverage among Asian American individuals between 2011 and 2018, with other factors remaining stable. Furthermore, race-specific associations have been observed between BMI levels and other cardiometabolic outcomes (eg, greater risk for Asian adults than White adults with the same BMI).^47,48,49^ In our adjusted analyses, the increasing BMI entirely explained the increasing serum urate trend among Asian individuals over the 8 years, but only partially explained the worsening gout prevalence. These findings suggest that, unlike serum urate trends, there are other factors contributing to the gout prevalence trend that were not included in our models. Furthermore, the effect period between elevation in serum urate concentrations and developing the actual clinical end point of gout may take more time than contemporaneous 2-year NHANES cycles. To that end, future studies with extended periods would be valuable to further trace trends and clarify the reasons behind the increase in gout prevalence among Asian individuals.

We found that the Asian vs White disparity in gout prevalence and serum urate concentrations in the NHANES 2017 to 2018 and those in the UKBB through 2021 increased after accounting for social determinants of health, lifestyle, and clinical factors. This is in striking opposition to the gout disparity observed among Black individuals, which was entirely explained by the same socioclinical and lifestyle factors^13^ (ie, nurture), whereas Asian disparities are likely associated with both nature (genetics) and nurture (Westernized environments). Indeed, individuals of Asian ancestry have a higher prevalence of *SLC2A9*, *ABCG2*, and other urate risk variants,^16,50^ which are key contributors to hyperuricemia and gout.^51^ Although genetic liability among Asian individuals could not have changed over 8 years or contributed to the increasing trend or created the ecological differences between Asian and Western countries, gene-environmental interactions (including unmeasured factors) could play a substantial role among genetically susceptible Asian populations. Indeed, recent studies^52,53^ have found and replicated substantial interactions between genetics and lifestyle and related factors such that the deleterious effect of genetic predisposition for gout is more pronounced among those with obesity or unhealthy diet.

Although the underlying mechanisms for these observations require further study, the disproportionate increase in prevalence among Asian individuals nonetheless has implications for gout clinical care as well as population health. For example, in a nationally representative analysis of 3.9 million ambulatory visits for gout, Asian individuals with gout were significantly less likely to receive a prescription for allopurinol, a highly effective urate-lowering agent that prevents recurrent gout flares,^54^ compared with their White counterparts.^55^ Furthermore, a recent nationwide study^56^ of US emergency department visits and hospitalizations also found that Asian adults experience excess rates of ED visits and hospitalizations with a primary diagnosis of gout compared with White adults, leading to avoidable health care utilization and costs. These findings call for more race-specific research in gout epidemiology, risk factors, and management strategies and interventions, including appropriate urate-lowering therapy combined with risk factor interventions, that are culturally appropriate to address these disparities.

### Limitations

This study has limitations that should be addressed. First, it was performed in a nationally representative sample of US adults; thus, the findings are likely to be generalizable to the entire US population. However, the UKBB population, which confirmed the Asian-White disparity observed in the US, is less representative of the UK general population.^57,58^ This may, in part, account for the lower gout prevalence in the UK than the US, although it is consistent with prior estimates.^5^ Nonetheless, the documented racial disparity should be generalizable, even if the participants are not representative of the population at large.^57,58^ In the NHANES, ascertainment of gout status by self-report of health care professional–diagnosed gout could have missed gout cases not diagnosed by health care professionals, potentially due to racial and ethnic differences in access to care. However, adjusting for active insurance status did not notably affect our trend analysis over time or disparity comparison in 2017 to 2018. Furthermore, we still found that Asian individuals had the highest and increasing prevalence of gout among Medicare-eligible Americans aged 65 years and older (with similar health care coverage), as well as in the UKBB where universal health care is available. Conversely, as gout ascertainment in the NHANES has not been validated against established criteria, to our knowledge, this method of gout ascertainment could overestimate gout prevalence related to health care professional misdiagnosis or patient misunderstanding. Nevertheless, the ascertainment method was the same as widely cited previous NHANES analyses,^7,32^ which allowed for direct comparisons over time even beyond the current trend analysis. In addition, our serum urate data corroborated gout prevalence data in the NHANES and UKBB.

Although this study was able to analyze US Asian individuals as a whole, this category encompasses more than 40 subgroups,^15^ and efforts to mitigate the rising burden of gout among Asian adults would benefit from subgroup-specific data,^15^ as numbers allow. Similarly, NHANES data do not allow examination of subgroups within Hispanic ethnicity (eg, Black Hispanic individuals).^37^ Further tracking of these national data as they become available would be valuable to confirm these trends and evaluate them in greater depth.

## Conclusions

Gout now affects more than 12 million US adults because of an aging population and increasing prevalence among Asian individuals, which numerically surpassed all other racial and ethnic groups in 2017 to 2018. This Asian vs White disparity does not appear to be associated with socioclinical factors.

## Notes

Supplement 1. **eAppendix.** Supplemental Methods
eReferences
**eTable 1.** Prevalence of Gout and Hyperuricemia in the US, 2017-2018 **eTable 2.** Trends in Mean Serum Urate: NHANES 2011-2018 **eTable 3.** Trends in Characteristics of Asian and White Adults from NHANES 2011 to 2018 **eTable 4.** Prevalence of Gout and Hyperuricemia by Racial/Ethnic Group Standardized to the UK General Population **eTable 5.** Characteristics of UK Biobank Participants, by Race **eFigure.** Trends in Gout Prevalence Among US Adults 65 Years and Older by Race/Ethnicity: NHANES 2011-2018

Supplement 2.
Data Sharing Statement
