# Supplementary material for: Trends in Prevalence of Gout Among US Asian Adults, 2011-2018
Source: JAMA Netw Open. 2023 Apr 21;6(4):e239501. doi: 10.1001/jamanetworkopen.2023.9501 (PMC10122173; doi:10.1001/jamanetworkopen.2023.9501)
Supplement: Supplement 1. — eAppendix. Supplemental Methods eReferences eTable 1. Prevalence of Gout and Hyperuricemia in the US, 2017-2018 eTable 2. Trends in Mean Serum Urate: NHANES 2011-2018 eTable 3. Trends in Characteristics of Asian and White Adults from NHANES 2011 to 2018 eTable 4. Prevalence of Gout and Hyperuricemia by Racial/Ethnic Group Standardized to the UK General Population eTable 5. Characteristics of UK Biobank Participants, by Race eFigure. Trends in Gout Prevalence Among US Adults 65 Years and Older by Race/Ethnicity: NHANES 2011-2018 [file jamanetwopen-e239501-s001.pdf]

# Supplemental Online Content

Yokose C, McCormick N, Lu N, et al. Trends in prevalence of gout among US Asian adults, 2011-2018. *JAMA Netw Open*. 2023;6(4):e239501.
doi:10.1001/jamanetworkopen.2023.9501

**eAppendix.** Supplemental Methods
**eReferences**
**eTable 1.** Prevalence of Gout and Hyperuricemia in the US, 2017-2018
**eTable 2.** Trends in Mean Serum Urate: NHANES 2011-2018
**eTable 3.** Trends in Characteristics of Asian and White Adults from NHANES 2011 to 2018
**eTable 4.** Prevalence of Gout and Hyperuricemia by Racial/Ethnic Group Standardized to the UK General Population
**eTable 5.** Characteristics of UK Biobank Participants, by Race
**eFigure.** Trends in Gout Prevalence Among US Adults 65 Years and Older by Race/Ethnicity: NHANES 2011-2018

This supplemental material has been provided by the authors to give readers additional information about their work.

© 2023 Yokose C et al. *JAMA Network Open.*

**eAppendix. Supplemental Methods.**

**National Health and Nutrition Examination Survey (NHANES) Study Population**

The NHANES assesses a representative sample of the non-institutionalized US civilian population that is selected using a multistage, stratified sampling design. The survey is conducted continuously with data released in two-year cycles. It combines interviews, physical examinations, and laboratory data (https://www.cdc.gov/nchs/nhanes/about_nhanes.htm). After a home interview, participants attended examination sessions in which anthropometric measurements and blood specimens were obtained for laboratory assays.

**Assessment of Race and Ethnicity in NHANES**

Race and ethnicity were based on self-report and categorized as non-Hispanic White (hereafter referred to as White), non-Hispanic Black or African American (hereafter referred to as Black), Hispanic (Mexican and non-Mexican Hispanic), non-Hispanic Asian (persons having origins in any of the original peoples of the Far East, Southeast Asia, or Indian subcontinent, hereafter referred to as Asian), and Other, including American Indian or Alaskan Native, Native Hawaiian/ Pacific Islander, other, or more than one race. Per the NHANES analytic guidelines, all participants self-reporting Hispanic ethnicity were categorized as Hispanic, regardless of race, while participants self-reporting non-Hispanic ethnicity were categorized according to their reported race.(1) Asian race encompassed persons having origins in any of the original peoples of the Far East, Southeast Asia, or Indian subcontinent, including Cambodia, China, India, Japan, Korea, Malaysia, Pakistan, the Philippine Islands, Thailand, and Vietnam;(2) however, data on specific country or region of origin were not available.

**Assessment of Covariates in NHANES**

During the interviews, participants reported their sex, age, race/ethnicity, educational attainment, household size and income, health insurance, recent dietary intake, medical history, and use of prescription medications within the past 30 days (including diuretics). Household income and size were used to determine the poverty-to-income ratio, a derived variable used in prior NHANES studies(3-5) and calculated as household income, adjusted for household size, divided by the year-specific poverty guideline(6) issued by the US Department of Health and Human Services. Alcoholic beverage consumption was computed from responses to questions on the frequency of alcoholic beverage consumption and total number of drinks per day of consumption. Two 24-hour dietary recalls were undertaken to ascertain the types and amounts of all foods and beverages consumed by participants. After mapping to the US Department of Agriculture Food Patterns Equivalents Database,(7) these data were used to compute a diet quality score based on the dietary components emphasized or minimized in the Dietary Approaches to Stop Hypertension (DASH) diet.(8-10)

At the mobile examination units, participants had their height and weight measured by trained staff using standardized instruments, with body mass index (BMI) calculated by dividing the weight in kilograms by the square of the height in meters. Blood was drawn for measurement of concentrations of serum urate, creatinine, and other biomarkers. Serum creatinine concentrations were used to determine glomerular filtration rate (eGFR)(11) and chronic kidney disease (CKD) status, defined as eGFR $< 60$ mL/min.

**UK Biobank Study Population**

The UK Biobank (UKBB) resource is a prospective cohort of more than 500,000 residents of the United Kingdom (UK) aged 40 to 69 years at the time of enrollment (years 2006 to 2010) when participants provided blood samples and attended a baseline visit.

**Assessment of Race in UKBB**

Self-reported ethnic group was categorized as Asian (Asian or Asian British, Chinese, Indian, Pakistani, Bangladeshi, or any other Asian background), Black (African, Black or Black British, Caribbean, or any other Black background), White (British, Irish, White, or any other White background), Mixed, and Other ethnic group. Although the term ‘ethnic group’ is used in place of ‘race’ in the UK,(12) ‘race’ is used in describing both the US and UK samples to avoid confusion with the distinct US categories of Hispanic and non-Hispanic ethnicity.

**Assessment of Gout and Hyperuricemia in UKBB**

The UK Biobank obtained ethical approval from the North West - Haydock Research Ethics Committee (16/NW/0274), and all participants provided written informed consent, including consent for their data to be linked retrospectively and prospectively to primary care, hospitalisation, and death records.(13) This allowed for the ascertainment of gout cases from ICD and Read codes recorded in the linked hospitalisation, primary care, and death records data, as well as diagnoses reported by participants during the in-person assessments. Serum urate was

measured from each participant's baseline blood sample using an enzymatic assay on a Beckman Coulter AU5800 platform.(14)

**Assessment of Covariates in UKBB**

At the baseline visit, information was obtained through a touchscreen questionnaire and in-person interview on sociodemographics (including educational attainment), diet and lifestyle factors, and medical conditions and medications taken on a regular basis (including diuretics); anthropometrics, including height and weight, were also obtained by assessment center staff using calibrated instruments. Townsend deprivation index, a measure of area-level socioeconomic status derived from national census data, was assigned according to each participant's postcode of residence. BMI was computed by dividing the weight in kilograms by the square of the height in meters. Alcoholic beverage consumption was computed from responses to questions on the frequency of alcoholic beverage consumption, with six categories ranging from Never to Daily. Diet quality was computed from responses to questions on weekly consumption of red and processed meats, fish, and poultry, and consumption of milk. As with urate, creatinine was measured from each participant's baseline blood sample using an enzymatic assay on a Beckman Coulter AU5800 platform.(14) Serum creatinine levels were used to determine glomerular filtration rate (eGFR)(11) and chronic kidney disease (CKD) status, defined as eGFR <60 mL/min.

**UKBB Statistical Analysis**

All analyses using the UKBB data were conducted using R-studio. UK Census data(15, 16) were used to standardize prevalence to the UK general population within the age range of UKBB participants in 2021. Step-by-step logistic and linear regressions were undertaken to determine Asian-White differences in gout prevalence and serum urate concentrations, although active health insurance was not included due to the nature of the universal, publicly funded healthcare system in the UK.

© 2023 Yokose C et al. *JAMA Network Open.*

**eReferences.**


1. Chen TC, Clark J, Riddles MK, Mohadjer LK, Fakhouri THI. National Health and Nutrition Examination Survey, 2015-2018: Sample Design and Estimation Procedures. Vital Health Stat 2. 2020(184):1-35.
2. 2018;Pageshttps://wwwn.cdc.gov/nchs/data/nhanes/analyticguidelines/11-16-analytic-guidelines.pdf.
3. Abdalla SM, Yu S, Galea S. Trends in Cardiovascular Disease Prevalence by Income Level in the United States. JAMA Netw Open. 2020;3(9):e2018150.
4. Myers CA, Mire EF, Katzmarzyk PT. Trends in Adiposity and Food Insecurity Among US Adults. JAMA Netw Open. 2020;3(8):e2012767.
5. Zhang YB, Chen C, Pan XF, Guo J, Li Y, Franco OH, et al. Associations of healthy lifestyle and socioeconomic status with mortality and incident cardiovascular disease: two prospective cohort studies. BMJ. 2021;373:n604.
6. U.S. Department of Health and Human Services 2021;Pageshttps://aspe.hhs.gov/topics/poverty-economic-mobility/poverty-guidelines.
7. US Department of Agriculture 2021;Pageshttps://www.ars.usda.gov/northeast-area/beltsville-md-bhnrc/beltsville-human-nutrition-research-center/food-surveys-research-group/docs/fped-overview/.
8. Fung TT, Chiuve SE, McCullough ML, Rexrode KM, Logroscino G, Hu FB. Adherence to a DASH-style diet and risk of coronary heart disease and stroke in women. Arch Intern Med. 2008;168(7):713-20.
9. Yokose C, McCormick N, Lu N, Joshi AD, Curhan G, Choi HK. Adherence to 2020 to 2025 Dietary Guidelines for Americans and the Risk of New-Onset Female Gout. JAMA Intern Med. 2022.
10. Rai SK, Fung TT, Lu N, Keller SF, Curhan GC, Choi HK. The Dietary Approaches to Stop Hypertension (DASH) diet, Western diet, and risk of gout in men: prospective cohort study. BMJ. 2017;357:j1794.
11. Inker LA, Eneanya ND, Coresh J, Tighiouart H, Wang D, Sang Y, et al. New Creatinine- and Cystatin C-Based Equations to Estimate GFR without Race. N Engl J Med. 2021;385(19):1737-49.
12. Statistics OfN 2009;Pageshttp://www.ons.gov.uk/ons/guide-method/census/2011/the-2011-census/2011-census-questionnaire-content/final-recommended-questions-2011---ethnic-group.pdf.
13. Caleyachetty R, Littlejohns T, Lacey B, Besevic J, Conroy M, Collins R, et al. United Kingdom Biobank (UK Biobank): JACC Focus Seminar 6/8. J Am Coll Cardiol. 2021;78(1):56-65.
14. Biobank U 2019;Pageshttps://biobank.ctsu.ox.ac.uk/crystal/ukb/docs/serum_biochemistry.pdf.
15. ;Pageshttps://www.nomisweb.co.uk/sources/census.
16. ;Pageshttps://www.scotlandscensus.gov.uk/.
17. Zhu Y, Pandya BJ, Choi HK. Prevalence of gout and hyperuricemia in the US general population: the National Health and Nutrition Examination Survey 2007-2008. Arthritis Rheum. 2011;63(10):3136-41.
18. Chen-Xu M, Yokose C, Rai SK, Pillinger MH, Choi HK. Contemporary Prevalence of Gout and Hyperuricemia in the United States and Decadal Trends: The National Health and Nutrition Examination Survey, 2007-2016. Arthritis Rheumatol. 2019;71(6):991-9.


© 2023 Yokose C et al. *JAMA Network Open.*

## eTable 1. Prevalence of Gout and Hyperuricemia in the US, 2017-2018*

| | Prevalence of gout, % (95% CI) | No of Adults with Gout, (millions) | Serum urate level, mean mg/dl (95% CI) | Prevalence of hyperuricemia†, % (95% CI) |
|---|---|---|---|---|
| **Overall** | 5.1 (4.2, 5.9) | 12.1 | 5.4 (5.3, 5.4) | 20 (19, 21) |
| **Sex** | | | | |
| Male | 7.1 (5.6, 8.6) | 8.1 | 6.0 (5.9, 6.1) | 21 (18, 23) |
| Female | 3.2 (2.3, 4,1) | 3.0 | 4.8 (4.7, 4.8) | 19 (17, 21) |
| **Age** | | | | |
| 20-39 | 0.9 (0.2, 1.5) | 0.8 | 5.2 (5.1, 5.4) | 17 (14, 21) |
| 40-59 | 4.7 (3.2, 6.2) | 3.9 | 5.3 (5.2, 5.4) | 16 (14, 18) |
| 60-79 | 10.6 (8.3, 12.9) | 6.2 | 5.6 (5.5, 5.7) | 26 (23, 30) |
| ≥ 80 | 11.9 (8.9, 14.9) | 1.3 | 5.7 (5.5, 5.9) | 33 (26, 41) |
| **Medicare** | | | | |
| Covered | 10.4 (8.0, 12.7) | 5.4 | 5.3 (5.2, 5.3) | 32 (28, 35) |
| Non-Covered | 3.6 (2.6, 4.6) | 6.7 | 5.8 (5.6, 5.9) | 17 (15, 18) |
| **Race/ethnicity** | | | | |
| Asian** | 5.9 (3.2, 8.7) | 0.8 | 5.5 (5.4, 5.7) | 24 (18, 29) |
| Hispanic | 3.0 (1.4, 4.7) | 1.2 | 5.3 (5.2, 5.4) | 18 (15, 21) |
| Non-Hispanic Black | 5.1 (3.7, 6.5) | 1.4 | 5.5 (5.4, 5.7) | 24 (22, 27) |
| Non-Hispanic White | 5.6 (4.2, 6.9) | 8.3 | 5.3 (5.2, 5.4) | 19 (17, 21) |
| Other | 3.7 (1.9, 5.5) | 0.4 | 5.5 (5.3, 5.7) | 25 (16, 33) |

*Data presented incorporated sample weights and adjusted for clusters and strata of the complex sample design of NHANES 2017-2018 to represent the US population of this latest cycle, as was done in prior NHANES studies for the same purpose.(17, 18) Age-adjusted data for comparing cycles are presented in Tables 1 and 3.

95% CI = 95% confidence interval. †defined as >7.0 mg/dL for males and >5.7 mg/dL for females

**Asian race encompassed persons having origins in any of the original peoples of the Far East, Southeast Asia, or Indian subcontinent, including Cambodia, China, India, Japan, Korea, Malaysia, Pakistan, the Philippine Islands, Thailand, and Vietnam.

© 2023 Yokose C et al. *JAMA Network Open.*

**eTable 2. Trends in Mean Serum Urate: NHANES 2011-2018***

| | Mean Serum Urate, mg/dL (95% CI) | | | | |
|---|---|---|---|---|---|
| | 2011-12 | 2013-14 | 2015-16 | 2017-18 | P for trend |
| **Overall** | 5.4 (5.3, 5.5) | 5.4 (5.4, 5.5) | 5.4 (5.2, 5.5) | 5.4 (5.3, 5.4) | 0.61 |
| Age- and sex-adjusted | 5.4 (5.3, 5.5) | 5.4 (5.4, 5.5) | 5.4 (5.3, 5.4) | 5.4 (5.3, 5.4) | 0.53 |
| **All Participants**** | | | | | |
| Asian[a] | 5.4 (5.3, 5.5) | 5.5 (5.4, 5.6) | 5.5 (5.3, 5.6) | 5.6 (5.5, 5.7) | 0.009 |
| Hispanic | 5.2 (5.2, 5.3) | 5.3 (5.2, 5.3) | 5.3 (5.2, 5.3) | 5.3 (5.2, 5.4) | 0.27 |
| Non-Hispanic Black | 5.6 (5.5, 5.7) | 5.6 (5.5, 5.7) | 5.5 (5.5, 5.6) | 5.6 (5.4, 5.7) | 0.56 |
| Non-Hispanic White | 5.4 (5.3, 5.5) | 5.4 (5.4, 5.5) | 5.4 (5.3, 5.5) | 5.3 (5.2, 5.4) | 0.26 |
| Other | 5.3 (5.2, 5.5) | 5.4 (5.1, 5.7) | 5.4 (5.2, 5.6) | 5.5 (5.3, 5.7) | 0.42 |
| **Males***** | | | | | |
| Asian[a] | 6.0 (5.9, 6.2) | 6.2 (6.1, 6.3) | 6.0 (5.9, 6.1) | 6.3 (6.2, 6.5) | 0.07 |
| Hispanic | 5.9 (5.8, 6.0) | 5.9 (5.8, 6.0) | 5.9 (5.8, 6.0) | 6.0 (5.9, 6.2) | 0.24 |
| Non-Hispanic Black | 6.0 (6.0, 6.2) | 6.1 (6.0, 6.2) | 6.1 (6.0, 6.2) | 6.1 (6.0, 6.3) | 0.74 |
| Non-Hispanic White | 6.1 (6.0, 6.2) | 6.1 (6.0, 6.2) | 6.1 (6.0, 6.2) | 6.0 (5.9, 6.1) | 0.83 |
| Other | 6.0 (5.6, 6.4) | 5.9 (5.6, 6.2) | 5.9 (5.7, 6.1) | 6.0 (5.7, 6.2) | 0.89 |
| **Females***** | | | | | |
| Asian[a] | 4.7 (4.7, 4.8) | 4.8 (4.7, 4.9) | 4.9 (4.7, 5.1) | 4.9 (4.8, 5.0) | 0.03 |
| Hispanic | 4.6 (4.5, 4.7) | 4.7 (4.5, 4.8) | 4.7 (4.6, 4.7) | 4.6 (4.5, 4.7) | 0.62 |
| Non-Hispanic Black | 5.1 (5.0, 5.3) | 5.0 (4.9, 5.2) | 4.93 (4.9, 5.0) | 5.02 (4.9, 5.2) | 0.13 |
| Non-Hispanic White | 4.8 (4.7, 4.9) | 4.8 (4.8, 4.9) | 4.8 (4.7, 4.8) | 4.7 (4.6, 4.8) | 0.07 |
| Other | 4.7 (4.5, 4.9) | 4.7 (4.3, 5.1) | 4.9 (4.7, 5.7) | 5.0 (4.8, 5.3) | 0.12 |

*Data are presented incorporating sample weights and adjusted for clusters and strata of the complex sample design of NHANES 2011-2018.
**Age- and sex-standardized serum urate (mg/dL)
***Age-standardized serum urate (mg/dL)
95% CI = 95% confidence interval.
[a]Asian race encompassed persons having origins in any of the original peoples of the Far East, Southeast Asia, or Indian subcontinent, including Cambodia, China, India, Japan, Korea, Malaysia, Pakistan, the Philippine Islands, Thailand, and Vietnam.

© 2023 Yokose C et al. *JAMA Network Open.*

**eTable 3: Trends in Characteristics of Asian and White Adults from NHANES 2011 to 2018**

| | Asian* | | | | | White | | | | |
|---|---|---|---|---|---|---|---|---|---|---|
| | **2011-12 (N=11,721,622)** | **2013-14 (N=12,202,246)** | **2015-16 (N=13,646,775)** | **2017-18 (N=14,126,963)** | **p-trend** | **2011-12 (N=148,900,127)** | **2013-14 (N=150,727,360)** | **2015-16 (N=149,817,513)** | **2017-18 (N=148,869,215)** | **p-trend** |
| Male, N/% (95% CI) | 5,411,662<br>46.2 (44.1, 48.2) | 5,607,212<br>45.6 (43.5, 48.4) | 6,368,295<br>46.7 (44.2, 49.1) | 6,450,464<br>45.7 (42.1, 49.2) | 0.93 | 71,929,682<br>48.3 (45.8, 50.8) | 72,724,098<br>48.2 (46.4, 50.1) | 72,579,477<br>48.4 (46.5, 50.4) | 71,519,006<br>48.0 (45.5, 50.6) | 0.98 |
| Age, mean (SD) | 44.6 (1.2) | 45.3 (1.0) | 45.0 (1.3) | 45.6 (0.8) | 0.52 | 49.4 (0.9) | 49.7 (0.5) | 50.1 (0.7) | 50.5 (0.7) | 0.31 |
| Active health insurance, N/% (95% CI) | 9,329,077<br>79.6 (76.1, 83.1) | 10,359,968<br>84.9 (81.4, 88.4) | 12,148,196<br>89.0 (84.2, 93.8) | 12,749,245<br>90.2 (87.0, 93.5) | <0.001 | 128,364,152<br>86.2 (83.5, 88.9) | 132,277,302<br>87.8 (85.3, 90.2) | 137,714,673<br>91.9 (89.2, 94.7) | 134,089,572<br>90.1 (85.4, 94.8) | 0.07 |
| Education: high school or less, N/% (95% CI)[a] | 3,262,757<br>27.8 (20.2, 35.4) | 3,060,492<br>25.1 (17.0, 33.2) | 3,985,526<br>29.2 (22.5, 35.9) | 3,943,839<br>27.9 (16.9, 39.0) | 0.89 | 45,203,403<br>30.4 (23.5, 37.2) | 47,649,004<br>31.6 (25.9, 37.4) | 43,352,276<br>28.9 (23.8, 34.1) | 50,056,922<br>33.6 (29.0, 38.3) | 0.60 |
| Poverty: family income-to-poverty-ratio < 1.3, N/% (95% CI)[a] | 2,324,203<br>19.8 (15.3., 24.3) | 1,929,824<br>15.8 (9.4, 22.2) | 2,909,594<br>21.3 (13.5, 29.1) | 1,844,722<br>13.1 (7.7, 18.4) | 0.38 | 26,052,176<br>17.5 (13.8, 21.2) | 26,391,258<br>17.5 (11.9, 23.2) | 18,439,574<br>12.3 (10.2, 14.4) | 19,610,660<br>13.2 (11.9, 14.5) | 0.01 |
| BMI, mean (SD) | 24.5 (0.2) | 24.8 (0.2) | 25.0 (0.1) | 26.2 (0.2) | <0.001 | 28.5 (0.3) | 29.1 (0.2) | 29.3 (0.3) | 29.8 (0.3) | 0.007 |
| No. drinks per week, mean (SD) | 2.0 (0.3) | 1.5 (0.3) | 1.7 (0.1) | 1.4 (0.2) | 0.13 | 4.6 (0.4) | 3.7 (0.2) | 4.1 (0.3) | 3.7 (0.2) | 0.07 |
| DASH score, mean (SD)[b] | 25.0 (0.3) | 25.3 (0.4) | 25.5 (0.3) | 25.1 (0.4) | 0.66 | 25.9 (0.2) | 26.6 (0.2) | 26.4 (0.2) | 27.0 (0.3) | 0.001 |
| Diuretic use, N/% (95% CI)[a] | 354,083<br>3.0 (1.6, 4.4) | 206,796<br>1.7 (0.9, 2.5) | 389,848<br>2.9 (1.5, 4.2) | 433,127<br>3.1 (1.3, 4.8) | 0.81 | 12,257,852<br>8.2 (6.8, 9.7) | 10,639,788<br>7.1 (5.4, 8.8) | 11,673,728<br>7.8 (6.6, 9.0) | 10,124,627<br>6.8 (5.4, 8.2) | 0.04 |
| CKD: eGFR <60 mL/min, N/%, (95% CI)[a] | 228,759<br>2.0 (0.7, 3.2) | 252,309<br>2.1 (1.0, 3.1) | 337,983<br>2.5 (0.2, 4.8) | 345,371<br>2.4 (0.9, 4.0) | 0.79 | 8,970,010<br>6.0 (4.3, 7.7) | 10,680,247<br>7.1 (6.2, 7.9) | 7,664,267<br>5.1 (3.9, 6.3) | 8,744,755<br>5.9 (4.5, 7.2) | 0.09 |

BMI=body mass index; CKD=chronic kidney disease; DASH=Dietary Approaches to Stop Hypertension; eGFR=estimated glomerular filtration rate
*Asian race encompassed persons having origins in any of the original peoples of the Far East, Southeast Asia, or Indian subcontinent, including Cambodia, China, India, Japan, Korea, Malaysia, Pakistan, the Philippine Islands, Thailand, and Vietnam.

© 2023 Yokose C et al. *JAMA Network Open.*

**eTable 4. Prevalence of Gout and Hyperuricemia by Racial/Ethnic Group Standardized* to the UK General Population**

| | Prevalence of gout, % (95% CI) | Serum urate level, mean mg/dl (95% CI) | Prevalence of hyperuricemia†, % (95% CI) |
|---|---|---|---|
| **Overall** | 2.9 (2.9 to 3.0) | 5.1 (4.8 to 5.4) | 14.0 (13.9 to 14.2) |
| **Sex** | | | |
| Male | 5.4 (5.3 to 5.6) | 5.9 (5.9 to 6.0) | 17.5 (17.2 to 17.8) |
| Female | 0.8 (0.8 to 0.9) | 4.4 (3.9 to 4.9) | 11.4 (11.2 to 11.5) |
| **Race/ethnicity** | | | |
| Asian[a] | 3.3 (2.9 to 3.7) | 5.3 (5.0 to 5.5) | 16.0 (15.1 to 17.0) |
| Black[b] | 2.5 (2.1 to 3.0) | 5.1 (4.7 to 5.6) | 15.4 (14.3 to 16.6) |
| White[c] | 2.9 (2.9 to 3.0) | 5.1 (4.8 to 5.4) | 13.9 (13.8 to 14.1) |

*All estimates are age-standardized to represent the UK population.
[a]Including Asian or Asian British, Chinese, Indian, Pakistani, Bangladeshi, or any other Asian background; [b]Including African, Black or Black British, Caribbean, or any other Black background;
[c]Including British, Irish, White, or any other White background.

© 2023 Yokose C et al. *JAMA Network Open.*

**eTable 5: Characteristics of UK Biobank Participants, by Race***

| | Asian<br>N=9826 | White<br>N=407,057 |
|---|---|---|
| Age, mean (SD) | 65.4 (8.2) | 69.3 (8.0) |
| Education: high school or less, % | 35 | 43 |
| Deprivation score, mean (SD)[a] | 0.18 (3.2) | -1.51 (3.0) |
| Alcohol consumption | | |
| Daily, % | 6.5 | 21 |
| Never, % | 42 | 6.4 |
| Diet quality | | |
| Fish intake, servings/week, mean (SD) | 1.8 (2.1) | 2.2 (1.6) |
| Poultry intake, servings/week, mean (SD) | 1.8 (1.7) | 1.9 (1.3) |
| Meat intake, servings/week, mean (SD) | 2.7 (2.9) | 3.5 (2.3) |
| BMI, mean (SD) | 26.7 (4.4) | 27.3 (4.7) |
| Diuretic use, % | 4.9 | 6.4 |
| CKD: eGFR <60 mL/min, % | 1.2 | 1.3 |

***Participants with non-missing data on the seven socio-clinical factors included in the step-by-step regression analysis.
[a] Continuous variable, Townsend deprivation index (higher scores indicate a greater degree of neighborhood deprivation)

© 2023 Yokose C et al. *JAMA Network Open.*

**eFigure. Trends in Gout Prevalence* Among US Adults 65 Years and Older by Race/Ethnicity: NHANES 2011-2018****

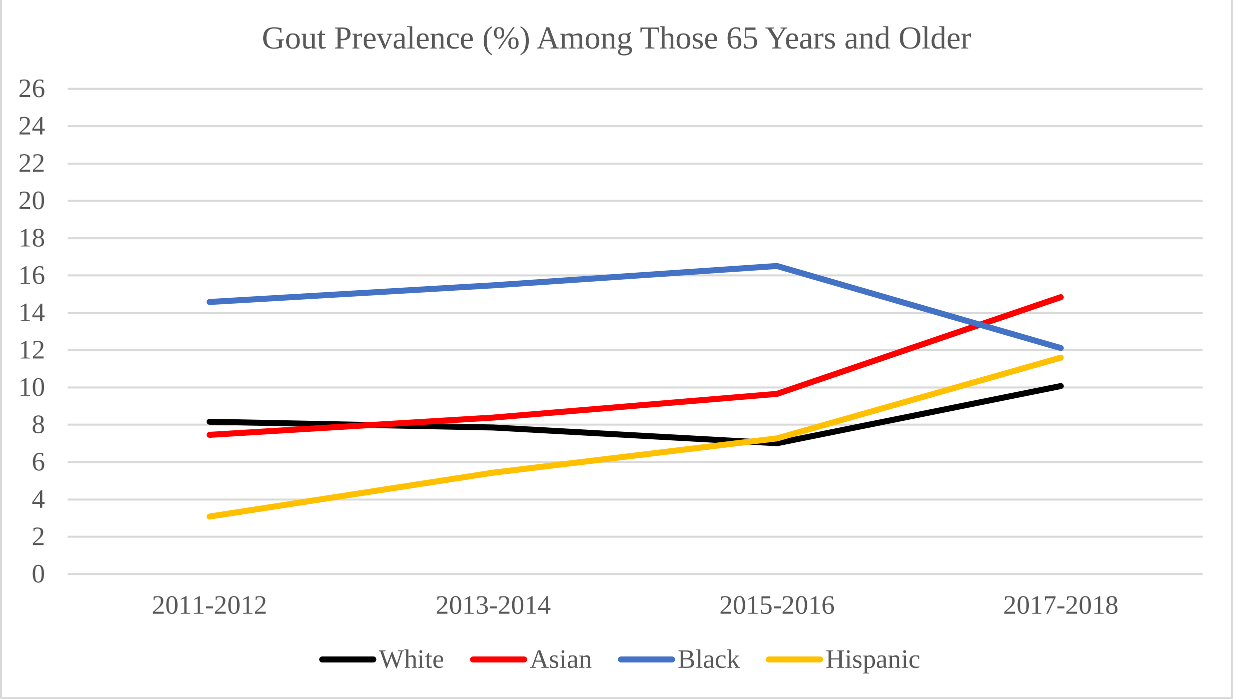


*Age- and sex-standardized prevalence
**Data are presented incorporating sample weights and adjusted for clusters and strata of the complex sample design of NHANES 2011-2018.

© 2023 Yokose C et al. *JAMA Network Open.*
