# Supplementary material for: Trends in Prevalence of Gout Among US Asian Adults, 2011-2018
Source: JAMA Netw Open. 2023 Apr 21;6(4):e239501. doi: 10.1001/jamanetworkopen.2023.9501 (PMC10122173; doi:10.1001/jamanetworkopen.2023.9501)
Supplement: Supplement 2. — Data Sharing Statement [file jamanetwopen-e239501-s002.pdf]

Data Sharing Statement

Yokose. Trends in Prevalence of Gout Among US Asian Adults, 2011-2018. *JAMA Netw Open*. Published April 21, 2023. doi:10.1001/jamanetworkopen.2023.9501

Data
Data available: No
